# Preliminary study on buffy coat smear and molecular detection of microfilaria in domestic chickens (*Gallus gallus domesticus*) raised in Southern Thailand

**DOI:** 10.14202/vetworld.2024.888-894

**Published:** 2024-04-20

**Authors:** Pornchai Pornpanom, Kanpapat Boonchuay

**Affiliations:** 1 Akkhraratchakumari Veterinary College, Walailak University, 222 Thaiburi, Tha Sala, Nakhon Si Thammarat, 80160, Thailand; 2 Informatics Innovation Center of Excellence, Walailak University, Nakhon Si Thammarat, 80160, Thailand; 3 One Health Research Center, Walailak University, Nakhon Si Thammarat, 80160, Thailand

**Keywords:** Buffy coat smear, Chickens, Cytochrome c oxidase I, Microfilaria, Nested-polymerase chain reaction

## Abstract

**Background and Aim:**

Filarial nematode typically produces a larval stage (microfilariae) in the bloodstream of vertebrate hosts, where microfilariae reside in the blood or subcutaneous tissues. Filarial nematodes cause human diseases, such as river blindness and elephantiasis, which are widely studied. However, in avian species, they are overlooked because they are nonpathogenic. In Thailand, microfilaria can be found in wild birds and domestic chickens. Recently, an increase in the number of blood samples submitted to veterinary diagnostic laboratories may have increased the number of microfilariae. Therefore, knowledge about filarial species and reliable detection methods are important. Therefore, this study aimed to investigate the efficacy of buffy coat smear and polymerase chain reaction (PCR)-based methods for the detection of microfilaria in domestic chickens. In addition, parasites were identified using the sequence of the cytochrome c oxidase subunit 1 (*COX1*) gene.

**Materials and Methods:**

Giemsa-stained buffy coat smears from a previous study were reanalyzed. These available buffy coat smears were prepared from 55 domestic chickens raised as backyard free-ranging in Southern Thailand. Fifty-seven frozen genomic DNA extracted from chicken blood were used to detect the presence of the *COX1* gene in *Onchocercidae* nematodes. The nested PCR protocol for amplification of the OnchoCOI_R2-OnchoCOI_R2 fragment of the *COX1* gene was applied from a previous report. Sequences of *COX1* were analyzed to identify Onchocercidae nematodes and if they were single or mixed infections. We constructed Bayesian phylogenetics to identify parasites and assessment of the relationship between filarial nematodes in avian species and other vertebrate hosts.

**Results:**

Buffy coat smears from 15 samples revealed microfilaria. Of these 15 samples, only eight were positive for *COX1* nested-PCR amplification. The other two buffy coat-negative samples were also positive for nested-PCR. Sequencing of these 11 nested PCR-positive samples revealed that almost all of them were *Onchocercidae* nematodes. Bayesian phylogenetic analysis showed that chicken Onchocercidae spp. were grouped with other avian filarial nematodes. However, all chickens Onchocercidae spp. showed a double peak in the sequencing chromatogram, indicating mixed filarial infection (species or haplotypes). Therefore, no chicken Onchocercidae sequence was deposited on National Center for Biotechnology Information, GenBank.

**Conclusion:**

Giemsa-stained buffy coat smear was a reliable method for the detection of chicken microfilaria in routine veterinary diagnostic laboratories. Development of a new PCR-based method is necessary. This method may provide greater sensitivity and specificity of detection. In addition, the PCR method allowed us to access the genetic characteristics of nematodes, which helped us maximize our knowledge of nematodes. Further investigations, such as the pathogenicity of filarial nematodes in chickens and their potential vectors, are required.

## Introduction

Filarial nematodes are viviparous and are transmitted by hematophagous arthropods belonging to the families Simuliidae, Ceratopogonidae, Tabanidae, and Culidae [1–3]. In vertebrate hosts, these parasitic nematodes typically produce a larval stage (microfilariae) into the bloodstream, after which they reside in the blood or subcutaneous tissues [4, 5]. In vectors, microfilariae develop to the third larval stage before being transmitted into the vertebrate host [6]. Adult filarial nematodes reside in connective tissues, limb joints, body cavities, cardiovascular, pulmonary, lymphatic, and nervous systems [2, 7–10]. A total of 16 genera of filarial nematodes belonging to the family Onchocercidae are found in avian hosts: subfamilies Dirofilariinae, Onchocercinae, Splendidofilariinae, and Lemdaniinae [4, 11–13].

Filarial nematodes cause human diseases, such as river blindness and elephantiasis [14–16]. These parasites are also important in veterinary medicine because they cause diseases in dogs (heartworm disease and subcutaneous dirofilariasis) and cats (heartworm disease) as well as zoonosis, including *Dirofilaria immitis*, *Dirofilaria repens*, *Brugia malayia*, and *Brugia pahangi* [17, 18]. Filarial infections in avian species (avian filariasis) are generally overlooked because they are non-pathogenic, resulting in a lack of studies on their pathogenicity, effects on avian host fitness [19], and detection techniques. However, fatal filarial infection has been reported in red-billed blue magpies (*Urocissa erythrorhynchus*) [10]. In domestic chicken (*Gallus gallus domesticus*), filarial infection has been reported, which may have a negative impact [12].

Conventionally, the diagnosis of human filarial infection is based on the detection of circulating microfilariae using Giemsa-stained thick blood smears [18]. In avian species, microfilariae can be readily observed in blood smears [20], but identification of microfilariae to filarial species that solely use morphologic features is doubtful because they are highly similar in morphology and morphometry [2, 7]. Recently, molecular techniques have been proved sufficient to detect filarial nematodes, identify species, and perform phylogenetic analyses [6, 21]. However, the use of filarial nematodes infected in domestic chickens may not be sufficiently sensitive and specific for use in routine laboratory diagnosis.

In Thailand, microfilaria have been reported in both wild birds [22] and domestic chickens (*G. gallus domesticus*) [20, 23, 24]. Microfilaria infection rates in domestic chickens in Northern and Southern Thailand are low (4.56%) and 4.21%, respectively [23, 24]. Two forms of microfilariae have been found in Thai chickens [24]: (i) unsheathed microfilaria with a short body and hook-like tail and (ii) unsheathed microfilaria with a long body and pointed tail. Although there is some information on microfilariae infected in Thai domestic chickens, there is not enough information on filarial species, molecular characteristics, and high-sensitivity detection methods. Investigation of the molecular characteristics of microfilaria in Thai chickens may help to develop high-sensitivity and high-specificity parasite detection techniques. Furthermore, it may provide baseline information for further investigations of filarial nematodes in chickens.

The aim of this study was to investigate Giemsa-stained buffy coat smear and molecular detection of microfilaria infection in Thai domestic chickens raised in Southern Thailand. The phylogenetic relationship of filarial parasites will also be investigated. Note that buffy coat smears were reanalyzed in this study, which provided more information related to molecular detection and microfilaria molecular characteristics of domestic chickens. This report provides sufficient information for routine laboratory diagnosis of microfilaria in chickens. In addition, molecular characteristics will serve as a baseline for the further development of highly sensitive and highly specific detection methods.

## Materials and Methods

### Ethical approval

The Walailak University Institutional Animal Care and Use Committee (Approval number: WU-AICUC-64014 and WU-ACUC-65052) and the Institutional Biosafety and Biosecurity Committee (Approval number: WU-IBC-64-006) approved the study.

### Study period and location

This study was conducted from June 2021 to June 2022 in Southern Thailand (Nakhon Si Thammarat, Phattalung, and Surat Thani). Laboratory analysis was performed at Laboratory of Hematology, Akkhraratchakumari Veterinary College, Walailak University.

### Blood sample collection

Blood samples were collected from June 2021 to June 2022. Blood samples (1 mL) were drawn from the brachial veins of 57 free-ranging chickens raised in three southern Thailand provinces: Nakhon Si Thammarat (23 samples), Phattalung (25 samples), and Surat Thani (nine samples). An ethylenediaminetetraacetic acid (EDTA) anticoagulant tube was used for blood stowing. EDTA blood was kept in an icebox and then submitted to the Laboratory of Hematology, Akkhraratchakumari Veterinary College, Walailak University. Within 12 h of collection, buffy coat smears were prepared from blood samples.

Briefly, EDTA blood was placed in a hematocrit tube and centrifuged at 15,000× *g* for 5 min. Subsequently, the tube was cut at the buffy coat layer with a few erythrocytes layer. A buffy coat with a few erythrocytes was smeared onto the glass slide. The smears were allowed to air dry at 35°C–37°C (an electric fan can be used to accelerate the drying process). Air-drained buffy coat smears were fixed in absolute methanol for 1 min and stained with 10% Giemsa solution in phosphate buffer (pH 7.2) for 45 min. The remaining blood samples were frozen for further molecular analysis.

### Microscopic examination

There were 55 buffy coat samples available. These buffy coat smears were detected for existing microfilaria using light microscopy at 400× magnification for 100 fields and were repeated at 1000× magnification for 100 fields. Microfilariae were photographed for morphology screening using a microscope (Olympus BX43, Olympus, Tokyo, Japan) equipped with a digital camera (OlympusDP27, Olympus) and CellSens imaging software (version 1.18, Olympus).

### Nested polymerase chain reaction (PCR) for cytochrome c oxidase 1 *(COX1)*

A total of 57 blood samples were included for molecular detection of the *COX1* gene of filarial nematodes. These 57 EDTA blood samples were extracted for genomic DNA using the Blood Genomic DNA extraction mini kit (FavorPrep, Pingtung, Taiwan). The DNA was then stored at –20°C until further processing. For nested PCR amplification of the fragment of *COX1 (*900 bp) of Onchocercidae nematodes, the protocol was followed in a previous report [5], with some modifications of the thermocycling conditions. In short, the external primers OnchoCOI_F1 (5´-TTG TGG AAT GAC TTT TGG YAA T-3´)/OnchoCOI_R1 (5´-AAT CTT AAC AGC TCT AGG AAT AGC-3´) and the internal primers OnchoCOI_F2 (5´-CTG TTA ATC ATA AGA CTA TTG GTA CT-3´)/OnchoCOI_R2 (5´-CAG CAC TAA AAT AAG TAC GAG TAT C-3´) were used for amplification of *COX1* fragment of microfilaria, which is in the larval stage of Onchocercidae nematodes.

The thermocycling conditions for each PCR step were as follows: Pre-denaturation (95°C for 5 min), 35 cycles of denaturation (95°C for 1 min), annealing (primary step: 53°C for 1 min and nested step: 50°C for 1 min), and extension (72°C for 1 min), which was completed by the final extension at 72°C for 10 min. All products from the primary reaction were diluted with distilled water (1:10) before being used as a template in the nested reaction. A total volume of 20 μL of the PCR mix was prepared, which contained 10 μL of master mix (OnePCR™, Ultra, Bio-Helix, New Taipei City, Taiwan), 1 μL of each primer (10 μM), 6 μL of distilled water, and 2 μL of DNA template (concentration <25 ng/μL in most samples).

A non-template control was used to make sure there were no false positives. The positive control was sample AVC 09, which showed microfilaria in the buffy coat smear. We checked the PCR products using 1.5% agarose gel prepared from the Agarose Tablets (Bio-Helix). The bands at 850–900 bp were then extracted and purified using PCR Clean-Up and Gel Extraction Kit (PureDirex, Bio-Helix). Purified DNA was submitted to Macrogen (Seoul, South Korea) for Sanger sequencing for both forward and reverse strands.

### Sequence analysis and phylogenetics

We retrieved 11 sequences of filarial nematodes from this study. Forward and reverse strands were screened for noise sequencing, and the contig sequence was generated using BioEdit version 7.0.5.3 [25]. Two samples with weak DNA sequences (AVC31 and AVC33) were excluded from the sequence analysis. The contig sequences of the other nine samples were blast on GenBank, to check whether they are Onchocercidae nematodes or not. Filarial nematode mixed infection was also identified using the double peak in the sequencing chromatogram.

Of these nine sequences, only one sequence (AVC09) exhibited a few noises. Therefore, it was used for further analysis. Because the similitude values in BLAST were low, 28 *COX1* sequences of Onchocercidae nematodes and other filarial nematodes from NCBI GenBank were included for phylogenetic analysis. The list of sequences follows a previous report Binkienė *et al*. [4]. We used the *COX1* sequence of *Ascaridia galli* (KT613888) as the out group.

Bayesian phylogenetic analyses of filarial nematodes were performed using MrBayes version 3.2.6, https://nbisweden.github.io/MrBayes/download.html [26]. The general time-reversible phylogenetic model was selected using the mrModeltest 2.3 program, https://github.com/nylander/MrModeltest2 [27] based on the hierarchical likelihood ratio test. The Markov chain Monte Carlo has been run for 3 million generations, with sampling every 100 generations. The first 25% of the samples were discarded as “*burn-*in.” Next, the consensus tree was calculated using the remaining 22,500 trees. Figtree version 1.4.3, http://tree.bio.ed.ac.uk/software/figtree/ visualized the tree. The genetic distance between sequences was calculated using the Jukes–Cantor model, in which all substitutions were equally weighted (implemented in MEGA version 11, https://www.megasoftware.net/) [28].

### Statistical analysis

The microfilaria infection rate was calculated on the basis of buffy coat smear examination results. Fisher’s exact test was used to assess differences in the microfilaria infection rate in each province (Nakhon Si Thammarat, Phatthalung, and Surat Thani). Fisher’s exact test was implemented in R version 4.2.2; p < 0.05 was considered statistically significant.

## Results

**Figure-1:**
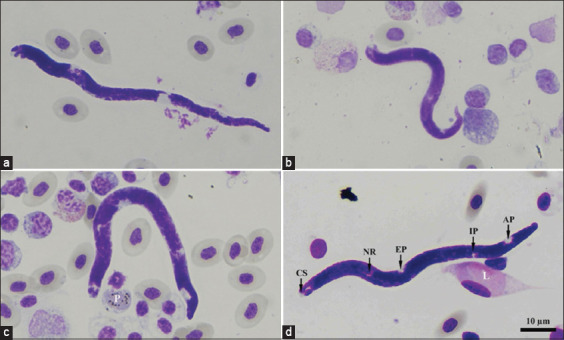
Microfilaria of Onchocercidae nematodes (a-d) in domestic chickens raised in Southern Thailand. Microfilaria had cephalic space (CS), nerve ring (NR), excretory pore (EP), inner body pore (IP) anal pore (AP). Noted, extracellular gametocytes of *Plasmodium* (P) and fusiform gametocytes of *Leucocytozoon* (L) were found in buffy coat smear. Giemsa staining.

Buffy coat smears revealed microfilariae in 15 chickens (Table-1). These microfilariae distinguish the cephalic space, nerve ring, excretory pore, inner body pore, and anal pore (Figure-1). The rate of infection was 27.27%. Nakhon Si Thammarat (14.54%), Phattalung (12.73%), and Surat Thani (0.00%) did not differ significantly in terms of microfilaria infection. Of these 15 samples, nested-PCR amplification of the cytochrome c oxidase subunit 1 (*COX1*) gene was successful in only eight samples. Furthermore, two buffy coat smear-negative samples were successfully used for *COX1* amplification. A total of 11 nested-PCR-positive samples revealed noisy sequencing for sequence analysis. Two of these sequences showed weak sequencing, which was excluded from sequence analysis. The other nine sequences were Onchocercidae nematodes with low similitude values in GenBank BLAST, ≤90% identity.

**Table-1:** Buffy coat smear and nested-PCR detection of microfilaria in backyard chickens (*Gallus gallus domesticus*) raised in Southern Thailand.

Chickens	Buffy coat smears (n = 55)	Nested-PCR (n = 57)	Sequencing
AVC07	Positive^a^	Negative	-
AVC09	Positive^b^	Positive	*Onchocercidae* spp.
AVC11	Positive^b^	Positive	*Onchocercidae* spp.
AVC12	Positive	Positive	*Onchocercidae* spp.
AVC13	Positive^b^	Negative	-
AVC14	Positive	Positive	*Onchocercidae* spp.
AVC15	Positive^b^	Positive	*Onchocercidae* spp.
AVC17	Positive^a^	Positive	*Onchocercidae* spp.
AVC25	Negative	Positive	*Onchocercidae* spp.
AVC26	Negative	Positive	*Onchocercidae* spp.
AVC31	Positive^b^	Positive	Weak sequencing
AVC32	Negative	Positive	*Onchocercidae* spp.
AVC33	Positive^b^	Positive	Weak sequencing
AVC36	Positive^b^	Negative	-
AVC38	Positive^b^	Negative	-
AVC39	Positive^c^	Negative	-
AVC44	Positive^b^	Negative	-
AVC45	Positive^b^	Negative	-

Noted, buffy coat smear examination revealed co-infection with other blood parasites: (a) Microfilaria, *Leucocytozoon* and *Trypanosoma*, (b) *Microfilaria* and *Leucocytozoon*, and (c) Microfilaria, *Leucocytozoon* and Plasmodium. Molecular characteristics of some *Leucocytozoon* and *Plasmodium* were reported by Boonchuay* et al*. [29]. PCR=Polymerase chain reaction

**Table 2:** Sequence of cytochrome c oxidase 1 of microfilaria isolated from chickens AVC09.

Host	Sequence
Chickens raised in Nakhon Si Thammarat, Thailand	CTATTTTGTCTATAATTATACGTTTTGAATTGTCTAGACCTGGTGGTTATTTGTTTTTTGG
	T AGCGGACAGGTGTATAATTCTGTTTTAACTATGCATGGTGTTTTAATAATTTTTTTTATGG
	T TATGCCAATTTTAATTGGTGGTTTTGGTAATTGAATGTTGCCTATTATATTGGGGGCTCC
	TG AAATAGCTTTTCCTCGTGTTAATGCGTCTATCTTTTTGATTTACTTTTGTAGCTTTAATGA
	T GGTTTATCAATCTTTTTTTATTGGCGGTGGACCTGGTAGAAGTTGAACTTTTTATCCTCC
	TT TGAGAGTGGAAGGTCAACCTGAAATATCTTTAGATGTTATAATTTTAGGTTTGCACACTG
	TA GGTATTGGTTCTTTATTGGGCGCTCTTAATTTTATAGTTACTGTTCAAAATATACGTTGTA
	A CACTGTTACATTAGATCAAGTTAGTATATTTGTTTGAACTTCTTATTTAACTTCTTTTTTAT TAGTTTTATCTATTCCTGTTTTAGCTGGTTCTCTTTTGTTTTTATTATTAGATCGTAATTTT AATACTTCTTTTTACGATACTAAAAAAGGAGGTAATCCTTTACTTTATCAACATTTGTTTT
	G ATTTTTTGGTCATCCTGAAGTTTATGTTATTATTTTACCTGTTTTTGGTATTATCAGAGAG
	G CTGTTTTATTTTTAACTGATAAGGATCGTTTGTTTGGTCAAACTAGAATAACTTTCTGCTT
	C TATTTGAATTGCTGTTTTAGGTACTTCTGTTTGAGGTCAT

**Figure-2:**
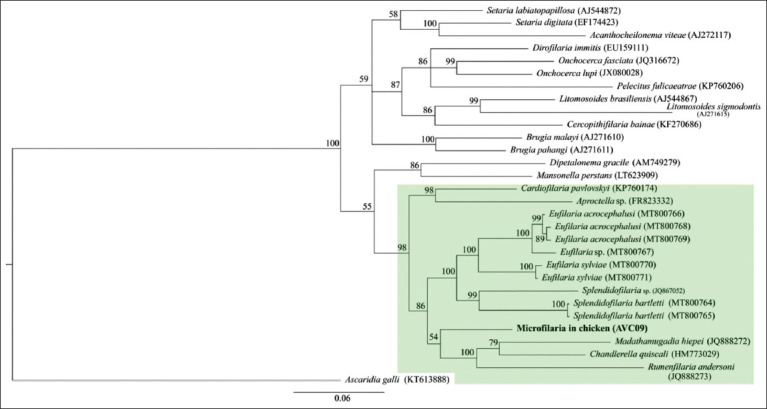
Bayesian phylogeny of Onchocercidae spp. (AVC09), given in bold, and other filarial nematodes. The consensus sequence of cytochrome c oxidase subunit 1 (*COX1*) was 630 bp. Avian filarial nematodes were grouped together (highlighted), with two filarial nematodes isolated from *Pachydactylus turneri* (JQ888272) and *Rangifer tarandus* (JQ888273). *COX1* sequence of *Ascaridia galli* (KT613888) was used as out group.

These nine sequences were identified as filarial nematode mixed infection due to the noise of the sequencing chromatogram (double peak chromatogram). We performed further phylogenetic analysis of one sequence with little noise (AVC09) to identify the possible genus of the parasite. The Bayesian phylogeny revealed that the *COX1* sequences of microfilaria AVC09 were grouped with those of other avian Onchocercidae nematodes (Figure-2.). This clade contained avian Onchocercidae nematode sequences and nematodes isolated from *Pachydactylus turneri* (JQ888272) and *Rangifer tarandus* (JQ888273). The genetic distance between *COX1* in filarial nematodes in avian hosts ranged from 0.00% to 16.89%. Microfilaria AVC09 is closely related to *Eufilaria sylviae* 923E (MT800771, 90.22% homology) and *E. Sylviae* 911E (MT800770, 90.02% homology). Both phylogenetic analysis and genetic distance showed that microfilaria found in chickens belong to the family Onchocercidae. However, the sequence did not contain a single peak in the chromatogram, which was considered to indicate mixed infection (species or haplotypes). Therefore, the Onchocercidae AVC09 sequence was not deposited in NCBI GenBank.

## Discussion

The microfilaria infection rate in chickens raised in Nakhon Si Thammarat and Phatthalung was relatively high at 27.27%. This is similar to the previous report of microfilaria-infected native chicken in Southern Thailand (18.79%) [24], where the infection rate in the layer was 3.64% and in broilers was 0.29%. These two reports detected microfilaria using buffy coat smear methods. This is evidence that further investigation for maximizing knowledge of avian filarial nematodes in chickens, sample collection in native chickens, or hybrid chickens raised as backyard free-ranging can gain the opportunity to obtain microfilaria. In addition, the stained buffy coat smear method was considered to be a reliable method for routine laboratory diagnosis of microfilaria infection. In addition, this inexpensive method can be used to used to detect other blood parasites such as *Plasmodium*, *Leucocytozoon*, and *Trypanosoma* [29].

There have been a few reports of species identification of filarial nematodes in domestic chickens (*G. gallus domesticus*) in Southeast Asia [12], including *Paronchocerca* (*Bhalfilaria*) *badami*, *Cardiofilaria* (*Gallifilaria*) *mhowensis*, and *Cardiofilaria nilesi*. Adult worms of these filarial nematodes were found in the heart, except in the body cavity of *C. nilesi*. However, their nucleotide sequences are not available in the NCBI GenBank database. Thus, our isolated sequences (Table-1) [29] did not determine whether these three nematodes were present. Apart from these three filarial nematodes, wild jungle fowls (*Gallus* spp.), which are the ancestor of domestic chickens, can be infected by more filarial species [12], including *Pelecitus galli*, *C. nilesi*, *Lemdana latifi*, *Lemdana sonneretta*, and *Lemdana* (*Singhneretta*) *sonneretta*. These filarial nematodes may have the potential to infect domestic chickens.

Although nested-PCR of *COX1* fragment OnchoCOI_F2-OnchoCOI_R2 works well in passerine birds [6], this protocol failed in some chicken blood samples, where buffy coat smear revealed microfilaria. This may be related to its sensitivity and specificity to chicken filarial nematodes. Another PCR protocol amplified the fragment COIintF-COIintR of *COX1* [30]. However, this protocol is likely to use touchdown PCR [4] because the thermocycler may not be available in some veterinary diagnostic laboratories. For these reasons, the design and development of a better PCR-based detection method is required. PCR detection not only shows infection status but also allows access to the genetic characteristics that help further parasite identification.

According to Bayesian phylogenetic analysis (Figure-2), our isolated sequences (AVC09) were grouped in avian Onchocercidae nematodes clade. This is evidence that the sequence of *COX1* can help in parasite identification. Information on nucleotide sequences and the morphological characteristics of adult worms or microfilaria should be interpreted. The morphological characteristics of microfilaria may not be used solely because they are highly similar in morphology and morphometry among filarial species [2, 7]. All our isolated sequences showed noise or double peak sequences that might indicate mixed filarial infection, which can be a mixed species or haplotype infection. Further investigations that combine both microscopic and molecular techniques are required. Filarial mixed infection can occur in chickens, which is supported by two morphotypes of microfilariae in chickens [24], namely, unsheathed microfilaria with short bodies and hook-like tails and unsheathed microfilaria with long bodies and pointed tails.

## Conclusion

This study revealed a high microfilaria infection rate (27.27%) in chickens raised as backyard free-ranging in Nakhon Si Thammarat and Phatthalung province. We found evidence that the staining-buffy coat smear method should be used for the detection of microfilaria in routine diagnostic laboratories. In addition, this report is the first molecular study of microfilaria-infected domestic chickens from Thailand. In this study, on the basis of the sequence of *COX1*, we identified *Onchocercidae* nematode infections that might be mixed infection (may be at the level of species or haplotypes). However, there is a need for the development of a new PCR-based detection method. This may increase the sensitivity and specificity of detection, as well as the characteristics of the *COX1* gene, which may help to identify parasites. Furthermore, we would like to encourage the study of filarial nematodes in domestic chickens in Southern Thailand. Further studies are necessary to better understand filarial nematode species, their pathogenicity, and their potential vector.

## Authors’ Contributions

PP: Conceptualization, methodology, investigation, data collection, writing-original draft preparation, writing-review and editing. KB: Data collection and data curation. Both authors have read, reviewed, and approved the final manuscript.
